# Important National Congress

**Published:** 1920-06-05

**Authors:** 


### Important National Congress.

### Wide Programme of Welfare Subjects.

The Congress of the Royal Sanitary Institute, which is to be held in Birmingham from July 19 to July 26, under the presidency of Viscount Astor, will be attended by about 1,500 delegates, including representatives from Government departments and many public bodies and societies. The Congress will be divided into various sections, and the skeleton programme is published in the *Birmingham Post*. The Sanitary Science and Preventive Medicine section will be presided over by Sir Robert H. Firth, and the subjects for discussion include "The falling birth-rate: Is it to be deplored?" and "Public health considerations relating to influenza, pneumonia, and allied epidemics." The Engineering and Architecture section will discuss "Housing," "Schools," Reconstruction of slum areas," and "Domestic drainage." Lieutenant-Colonel F. E. Fremantle, M.P., will take the chair in the Hygiene of Maternity and Child Welfare section, whose subjects include "The still-birth problem," and "The teaching of sex hygiene." The Personal and Domestic Hygiene section is to have as its principal topics for discussion "The choice and storage of suitable foods for the family." "The possibilities of personal and domestic hygiene in existing types of housing," and "The personal hygiene of the child" Mr. Neville Chamberlain, M.P., will preside over the Industrial Hygiene section for the consideration of "Industrial efficiency and fatigue," and "Welfare work in factories."

Representatives of Sanitary authorities will discuss "Open-air and tuberculosis schools, school camps, etc., and "Qualifications and training of health visitors. Dr. John Robertson (Medical Officer of Health in Birmingham) will preside over the conference of Medical Officers of Health, for the consideration of "The need for psychiatric clinics," and "The after-care of consumptives." The Engineers and Surveyors will deal with "Water supply," Arterial roads," "Sewerage," and "The hygiene of road pavements." Veterinary Inspectors will meet to consider "Diseases transmissible or disseminated by milk," "The neglect of local authorities to employ adequate staffs of veterinary and lay inspectors to prevent the public from consuming diseased, unsound, and unwholesome meat, milk, and fish," "The equipment and control of public abattoirs," and "The methods of spread and control of foot-and-mouth disease." Sanitary inspectors, health visitors, and others will also meet in conference.

This is altogether a good programme.

